# A Rare Cause and Alternative Algorithm for the Treatment of Gastrointestinal (GI) Bleed: Complications of a Failed Pancreatic Transplant

**DOI:** 10.7759/cureus.39741

**Published:** 2023-05-30

**Authors:** Soha Afzal, Madhu Mathew Vennikandam, Iftiker Ahmad, Radoslav Coleski, Dorian Jones

**Affiliations:** 1 Department of Internal Medicine, McLaren Greater Lansing, Lansing, USA; 2 Department of Internal Medicine, Michigan State University, Lansing, USA; 3 Division of Digestive and Liver Diseases, Sparrow Hospital, Lansing, USA; 4 Department of Gastroenterology and Hepatology, Sparrow Hospital, Lansing, USA; 5 Department of Gastroenterology and Hepatology, McLaren Greater Lansing Hospital, Lansing, USA

**Keywords:** gi bleed, gastrointestinal bleed, normocytic anemia, blood loss anemia, pancreas and kidney transplant, endovascular surgical repair, transplant failure complication, small bowel bleeding

## Abstract

A 39-year-old woman with no known risk factors presented for a recurrent upper gastrointestinal (GI) bleed. She had a prior history of failed kidney and pancreatic transplants secondary to childhood diabetes mellitus type I. After an extensive workup, she was found to have active hemorrhage into an area of the small bowel from an artery supplying her failed pancreatic transplant. Here, we discuss the importance of a systematic approach to evaluation, a high index of suspicion, and a known but not entirely common method of treatment for this condition.

## Introduction

Current recommendations for a patient with macrocytic anemia include an evaluation for myelodysplasia, bone marrow failure, hematologic malignancy, alcohol use, hemolysis, vitamin B12, or folate deficiency. If a patient simultaneously reports a symptom of melanotic stool, evaluation for an upper gastrointestinal (GI) bleed is warranted. Guidelines currently recommend that an esophagogastroduodenoscopy (EGD) be performed within 24 hours of a stable non-variceal suspected upper GI bleed [1]. With symptoms of hematochezia, it would be prudent to undergo an evaluation for a lower GI bleed. Guidelines recommend computed tomography (CT) angiography as the initial diagnostic test in patients with hemodynamically significant hematochezia [2]. Acute GI bleeding in pancreatic transplantation is a known but infrequent occurrence. They are often associated with the location of the transplantation and with arterioenteric fistulas. Angiography with vascular intervention was successful [3, 4].

We report the case of a patient with a history of failed kidney and pancreatic transplants who presented with melena and severe macrocytic anemia caused by an unusual source of bleeding. The most common vascular complication related to pancreatic transplants is allograft thrombosis, and arterial complications with bleeding have rarely been reported [5]. This case demonstrates the importance of a complete assessment of anemia with black, tarry stools and eventual transformation to frank hematochezia. This patient was found to have a small bowel bleed on imaging. The traditional algorithm of upper and lower endoscopy or subsequent small bowel enteroscopy may have provided relief for a patient with no prior history of organ transplantation. Our patient was provided with an alternative treatment that led to the complete recovery of her hematochezia. We emphasize that arterial bleeding in the setting of abdominal organ transplantation should be included in the differential GI bleed of any location and noted as a transplant complication. We also discuss an additional mode of management, not currently listed in general medical guidelines, that may be useful for a small bowel bleed.

## Case presentation

A 39-year-old woman was sent to the emergency room from her dialysis center after severe anemia was discovered. Her medical history was significant for renal and pancreatic transplants five years prior, postoperative graft failure one year prior, subsequent end-stage kidney disease on hemodialysis, anemia of chronic disease with baseline hemoglobin (Hgb) of 7-8 g/dL on intravenous (IV) iron and erythropoietin infusions, and insulin-dependent type I diabetes. Her solid organ transplants were the result of organ failure from childhood type I diabetes mellitus and were completed at an outside facility. On arrival at the emergency department, she was alert and oriented but was found to be drowsy on subsequent examination.

One week prior, she was admitted with macrocytic anemia and diabetic ketoacidosis (DKA). She denied using non-steroidal anti-inflammatory medications, oral iron supplements, anticoagulation, or bismuth subsalicylate. She denied symptoms of melena, hematochezia, or hematemesis. Lab work showed a Hgb of 4.1 g/dL, a mean corpuscular volume (MCV) of 109.5 fL, normal vitamin B12 and folate levels, platelets at 478 K/mcL, incalculable iron saturation and total iron binding capacity, ferritin at 4,215 ng/mL, transferrin at 152 mg/dL, normal fibrinogen, prothrombin time at 12.2 seconds, an international normalized ratio (INR) of 1.08, lactate dehydrogenase at 257 unit/L, and appropriately elevated reticulocytosis [Table 1].

**Table 1:** The patient's laboratory evaluation for hospital stays MCV: mean corpuscular volume; FLC: free light chain; INR: international normalized ratio

Labs	Initial presentation	Initial discharge	Subsequent presentation	Subsequent discharge	Normal values
Hemoglobin (g/dL)	4.1	8.4	3.1	7.5	12 - 15.5
MCV (fL)	109.5	99.6	108.4	99.6	83 - 96
Platelets (K/mcL)	478	331	245	356	150 - 350
Haptoglobin (mg/dL)	154				31.2 - 198.0
Absolute reticulocytes (x10^6/mcL)	0.13		0.14		0.03 - 0.08
Ferritin (ng/mL)	4,215				10 - 291
Iron level (UG/DL)	108				50 - 170
Lactate dehydrogenase (unit/L)	257		244		120 - 246
Total iron binding capacity (UG/DL)	Unable to calculate				228 - 460
Transferrin (mg/dL)	152				204 - 354
Iron saturation (%)	Unable to calculate				12.0 - 45.0
Vitamin B12 (pg/mL)	730		833		200 - 944
Folate (ng/mL)	>20.0				4.40 - 31.00
Fibrinogen (mg/dL)	288		217		200 - 500
Prothrombin time (seconds)	12.2		13.5	11.2	9 - 11.8
INR	1.08		1.21	0.99	0.9 - 1.10
Alpha 1 globulin (g/dL)	0.36				0.10 - 0.40
Alpha 2 globulin (g/dL)	0.71				0.60 - 1.00
Beta globulin (g/dL)	0.48				0.60 - 1.30
Gamma globulin (g/dL)	1.22				0.70 - 1.50
Kappa free light chain (FLC) (mg/dL)	31.27				0.33 - 1.94
Lambda free light chain (mg/dL)	23.52				0.57 - 2.63
Kappa/Lambda FLC Ratio	1.33				0.26 - 1.65
Albumin calculated (g/dL)	2.73				3.80 - 4.90

An EGD and colonoscopy one year prior at an outside facility for evaluation of anemia were unremarkable. A rectal examination was not performed. The gastroenterology consultants recommended continuing a hematological workup since there was no historical or observed overt bleeding. The hematology consultants added a myeloma panel, which showed no evidence of monoclonal paraproteins on immunofixation. Peripheral blood smears showed severe macrocytic anemia and reactive thrombocytosis. After an overall negative workup, the patient's anemia was deemed likely due to severe kidney disease. After four units of packed red blood cell (PRBC) transfusions, her anemia resolved, DKA was treated, and she was discharged home.

During her current presentation, there was no evidence of melena, hematemesis, or hematochezia. Vital signs showed hypotension (70/28 mmHg) with a mean arterial pressure of 43. Lab work showed a Hgb level of 3.1 g/dL, an MCV of 108.4 fL [Table 1], an anion gap of 30.2 nmol/L, a venous pH of 7.28, a glucose level of 503 mg/dL, beta-hydroxybutyrate greater than 46 mg/dL, sodium of 134 mmol/L, and a chloride level of 91 mmol/L. She was transfused with two units of packed red blood cells (PRBCs), received vasopressor support, and was infused with insulin. She was admitted to the intensive care unit (ICU), where she developed melena with further clinical deterioration. The melena transformed into multiple episodes of bright red blood per rectum during hemodialysis, requiring a mass transfusion protocol. She was started on intravenous (IV) pantoprazole, and the gastroenterology team was consulted.

**Figure 1:**
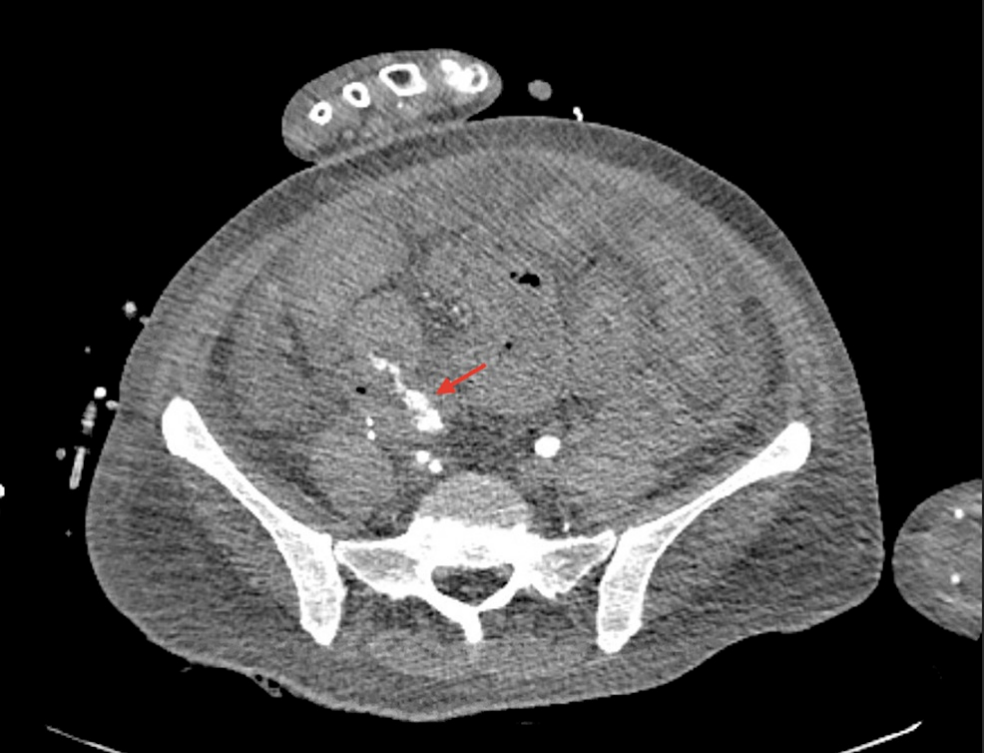
Extravasation of contrast dye in the loop of the small bowel (red arrow)

An emergent EGD was unremarkable. A computed tomography angiography (CTA) of her abdomen and pelvis was utilized as the next step to localize the source of her bleeding. CTA showed brisk contrast extravasation into a loop of distal small bowel from the artery that was supplying her failed pancreatic transplant [Figure 1].

**Figure 2:**
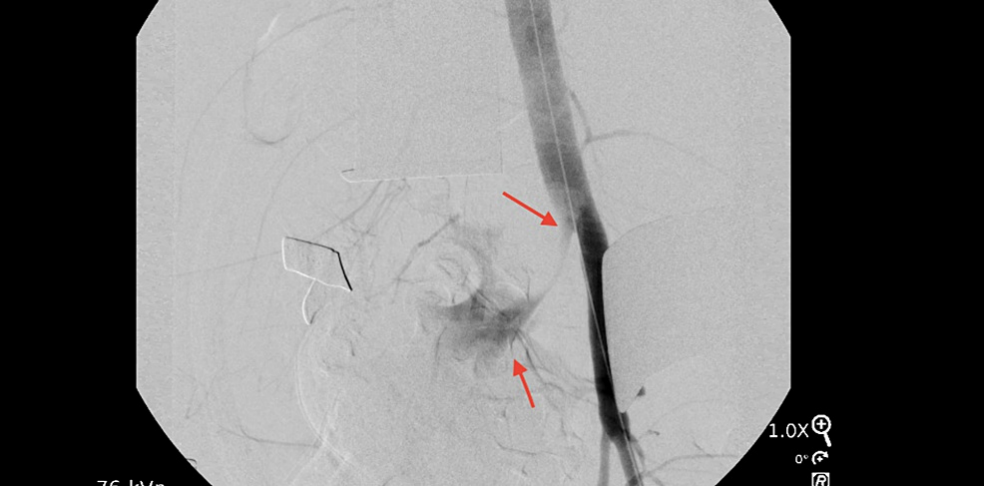
Extravasation of the vascular contrast dye out of the right common iliac artery (red arrow)

General surgery was consulted, and an emergent laparotomy was performed, which transformed into an open abdominal surgery. This revealed active bleeding from a branch of the right common iliac artery, supplying the failed pancreatic transplant. The patient was found to be hemorrhaging into the right lower abdomen and into the distal ileum, distal to the ligament of Treitz. Exploration noted dilation, fibrosis, and friability of the distal ileum and pancreatic transplant. It seemed that the bleed was occurring from the graft of the artery supplying the pancreatic transplant site, just distal to its anastomosis to the distal ileum [Figure 2].

At this point, the vascular surgery team was contacted for endovascular repair with subsequent small bowel resection of the area of the failed pancreatic transplant [Video 1].

**Video 1:** Post-stent angiography showing the resolution of contrast dye extravasation

Following surgery, the patient rapidly improved, and the rest of her hospital stay was uneventful. She was discharged home around 16 days later.

## Discussion

Hematochezia and melena are often used to distinguish between upper and lower GI sources of bleeding: hematochezia with the lower and melena with the upper [6]. An upper GI bleed is anatomically defined as occurring at a location proximal to the ligament of Treitz, while a lower GI bleed is distal [7]. This case was distinct in that the patient initially presented with melena but then later evolved to experience hematochezia. So where do we go from here? Multiple algorithms have been developed to evaluate a patient with a suspected upper GI bleed. After a negative EGD, the next step would be to undergo other studies to locate the source of the bleeding. Many of these algorithms recommend the early assistance of the GI team for endoscopic evaluation and possible intervention. However, in patients with hemodynamic instability, this may be easier said than done [1, 2, 8]. This patient was unique in that the location of her bleeding was from an artery supplying her failed pancreatic transplant flowing into her small bowel.

Complications related to dual pancreatic and kidney transplantation are noted to include posttransplant erythrocytosis, infection, pancreas failure, wound problems, and vascular thrombosis [9, 10, 11]. One study noted mortality related to upper GI bleeding in renal transplantation alone to likely be related to gastroduodenal ulcers in the setting of immunosuppressive therapy [12]. While another study mentioned a few instances of lower GI hemorrhage in renal transplantation alone due to ulcerating sigmoid and rectal disease [13], regarding pancreatic transplantation alone, one study mentions the development of arterial complications including pseudoaneurysm, arterial enteric-cystic fistula, or arteriovenous fistula. There have been multiple studies published that mention GI bleeding in pancreatorenal transplant patients but are also related to arterioenteric fistulas [3, 4]. Nonetheless, many of these studies reported endovascular management for the mentioned GI bleeds as well [3, 4, 14].

**Figure 3:**
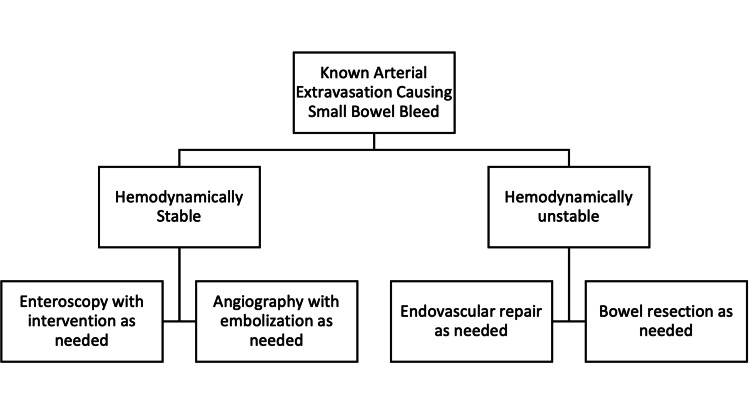
Algorithm for the treatment of small bowel bleed

Guidelines currently recommend initial management of general small bowel bleeding with endoscopic therapy. They mention that surgical therapy may be useful, and medical treatment with thalidomide or octreotide has shown some promise. Angiographic embolization can provide greater than 70% clinical success in retrospective studies. Vascular stenting is currently not listed in general medical guidelines as a recommended treatment modality for an arterial small bowel bleed [15]. One study evaluated 28 interventional radiologic treatments of arterioenteric fistulas in the setting of pancreatic transplantation. They found that vascular stenting is effective in controlling and preventing further bleeding [16]. Perhaps in patients with a medical history of pancreatic transplantation who present with an unknown cause of anemia and/or GI bleeding, the initial mode of treatment should be vascular intervention instead of endoscopic. Figure 3 shows a possible algorithm to be used for the treatment of small bowel bleeds. This may need to be explored further.

This patient’s initial presentation of melena and history of recurrent intermittent bleeding may have been due to the small, initial oozing of the branch of her right common iliac artery. The transition to frank, brisk hematochezia could have been due to hemodynamic changes related to hemodialysis, the administration of multiple blood products, and the incorporation of multiple vasopressors. Initially, prior to imaging, there was concern about an upper GI bleed that had transformed into a hemorrhage. This is what led to the early EGD. This patient’s hemodynamic instability was the limiting factor in undergoing CTA prior to an EGD. This may be a reason why there is a lack of data related to an arterial bleed from the supply of a failed pancreatic transplant. While it is unclear if the initial etiology of this patient's bleed was an arterioenteric fistula, this case adds to the limited literature on an additional complication of pancreatic transplant recipients. We also discussed a known yet medically alternative method of treating a small bowel bleed. A treatment that, while indicated in surgical guidelines, is not currently listed in the general medical guidelines [1, 3]. Lower GI bleeding must be kept as a differential diagnosis in this population with acute anemia, and the option of vascular stenting must be kept in the clinician’s toolbox early in the algorithm.

## Conclusions

This case represents an acute small bowel bleed likely secondary to a complication from a failed pancreatic transplant that was successfully treated with endovascular intervention. Complications related to dual pancreatic-kidney transplants or failure do not necessarily include the high likelihood of a vascular GI bleed. Regardless of the timing of transplant rejection, it is important to include GI bleeding in the differential diagnosis of a patient with a medical history of pancreatic transplant who presents with anemia.

Small bowel bleeds can be difficult to treat. Endoscopic treatment is the general medical mainstay of management in stable patients with no prior transplant history. However, in unstable patients with a history of pancreatic transplantation, endovascular management should be considered as an early option.
